# Environmental consequences of deltamethrin residues in cattle feces in an African agricultural landscape

**DOI:** 10.1002/ece3.3896

**Published:** 2018-02-14

**Authors:** Bryony Sands, Neludo Mgidiswa, Casper Nyamukondiwa, Richard Wall

**Affiliations:** ^1^ School of Biological Sciences University of Bristol Bristol UK; ^2^ Department of Biological Sciences and Biotechnology Botswana International University of Science and Technology Palapye Botswana

**Keywords:** agricultural ecosystem, cow dung, decomposition, dung beetle, paracoprid, pyrethroid, repellency, Scarabaeidae

## Abstract

Pyrethroid insecticides are widely used to control ectoparasites of livestock, particularly ticks and biting flies. Their use in African livestock systems is increasing, driven by the need to increase productivity and local food security. However, insecticide residues present in the dung after treatment are toxic to dung‐inhabiting insects. In a semiarid agricultural habitat in Botswana, dung beetle adult mortality, brood ball production, and larval survival were compared between untreated cattle dung and cattle dung spiked with deltamethrin, to give concentrations of 0.01, 0.1, 0.5, or 1 ppm. Cattle dung‐baited pitfall traps were used to measure repellent effects of deltamethrin in dung on Scarabaeidae. Dung decomposition rate was also examined. There was significantly increased mortality of adult dung beetles colonizing pats that contained deltamethrin compared to insecticide‐free pats. Brood ball production was significantly reduced at concentrations of 1 ppm; larval survival was significantly reduced in dung containing 0.1 ppm deltamethrin and above. There was no difference in the number of Scarabaeidae attracted to dung containing any of the deltamethrin concentrations. Dung decomposition was significantly reduced even at the lowest concentration (0.01 ppm) compared to insecticide‐free dung. The widespread use of deltamethrin in African agricultural ecosystems is a significant cause for concern; sustained use is likely to damage dung beetle populations and their provision of environmentally and economically important ecosystem services. Contaminated dung buried by paracoprid (tunneling) beetles may retain insecticidal effects, with impacts on developing larvae below ground. Lethal and sublethal effects on entire dung beetle (Scarabaeidae) communities could impair ecosystem function in agricultural landscapes.

## 1 INTRODUCTION

Ectoparasites of cattle (ticks, flies, and lice) and the diseases they transmit provide major production constraints in Africa (Ilemobade, 2009; Mukhebi & Perry, 1992). Treatment with insecticides is often considered necessary to prevent livestock and production losses, and their use in African livestock production is increasing driven by increased ectoparasite incidence with global change (Cumming & Van Vuuren, 2006) and the need to increase local food security (Nonga, Muwonge, & Mdegela, 2012). Pyrethroid formulations of deltamethrin and cypermethrin are widely recommended and used for biting fly and tick control (Alexander & Wardhaugh, 2001; Lovemore, 1992; Spickett & Fivaz, 1992).

The main route of excretion following treatment of cattle with pyrethroids is via the feces (Floate, Wardhaugh, Boxall, & Sherratt, 2005), and they have been shown to be present in dung at concentrations of about 0.01–0.4 ppm for up to 2 weeks after administration (Vale, Grant, Dewhurst, & Aigreau, 2004; Wardhaugh, Longstaff, & Lacey, 1998). Excreted unmetabolized drug or metabolites (Venant, Belli, Borrel, & Mallet, 1990) may retain insecticidal properties (Floate et al., 2005; Wardhaugh, 2005). Topical formulations appear to give the highest dung contamination, with 96%–98% of the eliminated dose present in cattle feces after treatment with a deltamethrin pour‐on product (Venant et al., 1990; Wardhaugh, 2005). Pyrethroids are not quickly degraded in dung; pats spiked with 10 ppm deltamethrin showed no change in concentration over 2 months of field exposure (Vale et al., 2004).

Dung beetles, particularly tunneling (paracoprid) species which comprise ~70% of the dung beetle species in Africa (Davis, Frolov, & Scholtz, 2008), are responsible for rapid dung removal from the soil surface (Hanski & Cambefort, 1991). They excavate tunnels underneath pats, and form brood balls with the dung below ground, in which they lay their eggs (Hanski & Cambefort, 1991). In contrast, in temperate climates earthworms play a major role in dung degradation (Holter, 1979), and dung beetle communities are characterized by endocoprid species that do not make tunnels but live within the dung pat itself (Hanski & Cambefort, 1991). Tunneling and dung burial by paracoprid beetles therefore have a vital role in semiarid ecosystems and have been shown to improve the physiochemical characteristics of the soil and increase feed value of herbage in terms of yield, nitrogen percentage, total crude protein, and digestible nutrient in grass shoots (Bang et al., 2005; Bertone, Green, Washburn, Poore, & Watson, 2006). Detrimental effects of insecticide residues on dung fauna, compounded by changes to functional assemblages in response to climate change, may result in loss of the vital ecosystem services they contribute to pastureland (Beynon, Wainwright, & Christie, 2015; Slade & Roslin, 2016).

To date, laboratory studies have shown toxic effects of dung contamination with a range of pyrethroid compounds on several dung beetle species, including both direct mortality and negative effects on reproduction (e.g., Bang, Lee, Na, & Wall, 2007; Bianchin, Alves, & Koller, 1998; Bianchin, Honer, Gomes, & Koller, 1992; Wardhaugh et al., 1998). Models suggest that a single deltamethrin treatment may cause up to 75% reduction in beetle activity by the end of a season (Wardhaugh et al., 1998) and that treatment at a frequency of once or twice per month may result in 10%–30% beetle mortality (Vale et al., 2004). Negative effects on dung beetle adult and larval stages have also been reported in field studies (Chihiya, Gadzirayi, & Mutandwa, 2006; Krüger, Scholtz, & Reinhardt, 1998; Mann, Barnes, Offer, & Wall, 2015; Vale et al., 2004). However, when dung contaminated with insecticide residues is buried by paracoprids, any detrimental effects on dung beetle juvenile stages take place beneath the ground and are not easily quantified. Despite their importance to ecosystem function, impacts of fecal insecticide residues on dung beetles in semiarid African landscapes are relatively understudied compared to temperate pasture systems.

The aims of this study were therefore to quantify the ecological impacts of the widely used topical pyrethroid deltamethrin in a semiarid African agricultural landscape, considering the whole dung beetle community but focusing in particular on paracoprid (tunneling) species which are dominant in this ecosystem. The study aimed to consider direct lethal effects in terms of adult mortality and sublethal effects on brood ball production, egg hatch, and larval development through mesocosm experiments. The attractiveness and decomposition rate of contaminated dung on pastures were also examined to give a wider picture of how this commonly used insecticide might impact dung beetle populations and ecosystem function in an agricultural system.

## 2 METHODS

### 2.1 Field site

The work was undertaken near Khumaga Village, Central District, Botswana (S20° 28.165′, E24° 30.875′)—a semiarid region with Kalahari sand soils, bordering the Boteti River on the western edge of the Makgadikgadi Pans. Experiments were carried out between December 2015 to March 2016 and January to March 2017. The area is characterized by small‐scale cattle (~16 LSU) and goat (~2.3 LSU) pastoralists. All experiments used an ungrazed, fenced plot, approximately 200 × 200 m. The vegetation type was acacia scrub, including camelthorn *Acacia erioloba* and blackthorn *Acacia mellifera*.

### 2.2 Dung

Freshly voided cattle dung was collected at 07:00 hr on the day of use from a herd of approximately 50 Tswana/Sanga‐type cattle that were corralled overnight but otherwise foraged freely during the day. The animals had never been treated with parasiticides, and all dung was homogenized by thorough mixing before being used. A commercial deltamethrin product (Butox^™^ Swish, MSD Animal Health, 0.75% w/v deltamethrin pour‐on suspension) was thoroughly mixed into feces to produce 10 kg batches of dung with concentrations of 0.01, 0.1, 0.5, and 1 ppm. For each concentration, the appropriate volume of deltamethrin was transferred to a beaker using a micropipette (Fisherbrand, Fisher Scientific, Leicestershire, UK) and made up to 100 ml with water. This was mixed thoroughly before being poured slowly into the dung while mixing, and the dung was homogenized by mixing for a further 3 min to ensure the insecticide was evenly distributed throughout. For the control batch, 100 ml of water only was mixed with the dung in exactly the same manner.

### 2.3 Adult mortality—Field experiments

To examine the effects of fecal residues of deltamethrin on adult beetles naturally colonizing dung in the field, mesocosms were created. These consisted of deep cylindrical buckets (60 × 45 cm) arranged in a grid, spaced 5 m apart and buried in the ground so that the rim of the bucket was flushed with the surface. The buckets were refilled with soil to within 2.5 cm of the top, creating a barrier that prevented telecoprid (ball rolling) beetles removing feces and moribund beetles from crawling away. In year 1, 50 pats, 10 of each of the four deltamethrin concentrations and 10 control pats, were formed using a 20‐cm‐diameter plastic pat former that held 1 kg feces. One pat was placed on the surface of the soil in the center of each bucket with treatments allocated to a position in the grid at random. In year 2, the 0.5 ppm concentration was omitted giving 40 buckets in total, because the magnitude of the effect was not significantly different from 1 ppm. Dead dung beetles were collected from the surface of the sand inside the buckets twice a day at 07:00 hr and 18:00 hr for 5 days; after this, time beetles were no longer found. After collection, beetles were stored in ethanol.

### 2.4 Adult mortality—Bioassays

For adult mortality assays, cow dung‐baited pitfall traps were used to collect an abundant, medium‐sized paracoprid beetle *Metacatharsius troglodytes* (Boheman 1857; ~10 mm, 43.5 mg dry wt (Davis, Scholtz, & Swemmer, 2012)). Beetles were stored in plastic containers (15 × 14 × 20 cm) ¼ filled with sand as a substrate, and covered with 0.5‐mm insect mesh until use, approximately 2 hr after collection. Fifteen plastic containers (15 × 14 × 20 cm) were ¼ filled with sand, and 150 g dung was placed on the surface in the center of each, forming three repeats of the five deltamethrin concentrations. Ten *M. troglodytes* were added to each container, and insect mesh (0.5 mm) was secured over the top to prevent beetles escaping. The containers were stored in a permanently shaded area, and beetles were checked for mortality every 24 hr for 10 days. Mortality was defined as prolonged absence of movement of the legs, antennae, or abdomen following gentle prodding with forceps.

### 2.5 Reproduction—Mesocosm experiments

Mesocosms composed of sand‐filled buried cylindrical buckets (60 × 45 cm), as described above, were used to examine brood ball production and larval development. Ten 1 kg artificial pats, each of three deltamethrin concentrations (0.01, 0.1, and 1 ppm) and ten noninsecticidal controls, were randomly allocated to be placed on the sand in each bucket and left in position for 14 days to allow natural colonization and brood ball production by dung beetles. Mesocosms were then lifted out of the ground, and the contents were hand‐searched for dung beetle brood balls, larvae, and pupae. Brood balls were counted and then opened to examine larval development inside. The total number of brood balls, larvae, pupae, and unhatched eggs per mesocosm was recorded.

### 2.6 Repellency—Field experiments

Repellent effects of deltamethrin residues in dung were investigated using cow dung‐baited pitfall traps. Traps were half‐filled with water and 0.5 ml detergent. In year 1, three pitfall traps baited with dung either containing no deltamethrin or deltamethrin at 0.1 and 1 ppm were set up 20 m apart in three different locations (separated by 200 m), giving a total of nine traps. In year 2, 12 pitfall traps were set up 10 m apart and baited with dung containing deltamethrin at 0.01 ppm, 0.1 ppm, and 1 ppm or untreated dung. Treatments were allocated to positions within the grid at random. Traps were set up at 07:00 hr and emptied the following morning at 07:00 hr; after collection, beetles were stored in ethanol.

### 2.7 Dung decomposition

The decomposition rate of dung containing deltamethrin was contrasted with that of control dung without added deltamethrin. Forty 1 kg artificial pats, spaced 5 m apart, were placed in a grid with 10 replicates of each of three deltamethrin concentrations (0.01, 0.1, and 1 ppm) and untreated controls. Treatments were allocated to positions within the grid at random. Pats were left exposed on the ground to natural conditions for 5 weeks, and on days 7, 14, 21, 28, and 35, all pats were lifted and weighed. They were then returned to their positions on the ground.

### 2.8 Statistical analysis

All statistical analysis was performed using RStudio (Version 1.0.44, RStudio Team 2016). Means are reported ±*SE* unless otherwise stated. For field experiments, a general linear model with a negative binomial error distribution (package “MASS”) was performed on count data of number of dead beetles, and number of brood balls, larvae, and unhatched eggs recovered, with insecticide concentration as the independent variable. For the number of pupae recovered, zero‐inflated count data regression was performed with a negative binomial error distribution.

For bioassays of *M. troglodytes*, logistic regression was performed on beetle mortality over time for exposure to each deltamethrin concentration. Time taken for 50% of the beetles to die (LT_50_) was calculated. An ANOVA was then used to compare the LT_50_ between the different deltamethrin concentrations.

To investigate any repellent effects of contaminated dung, a general linear model with a negative binomial error distribution was performed on count data of the number of beetles attracted to dung‐baited pitfall traps with insecticide concentration as the independent variable.

To compare dung decomposition rate between insecticide concentrations, a repeated measures linear mixed model (package “lme4”) with a Gaussian distribution was performed with dung weight as the dependent variable, deltamethrin concentration as a fixed effect, and the interaction term between deltamethrin concentration and number of days in the field (7, 14, 21, 28, and 35) included. Day number nested within individual pat was a random factor.

## 3 RESULTS

### 3.1 Adult mortality—Field experiments

**Figure 1:**
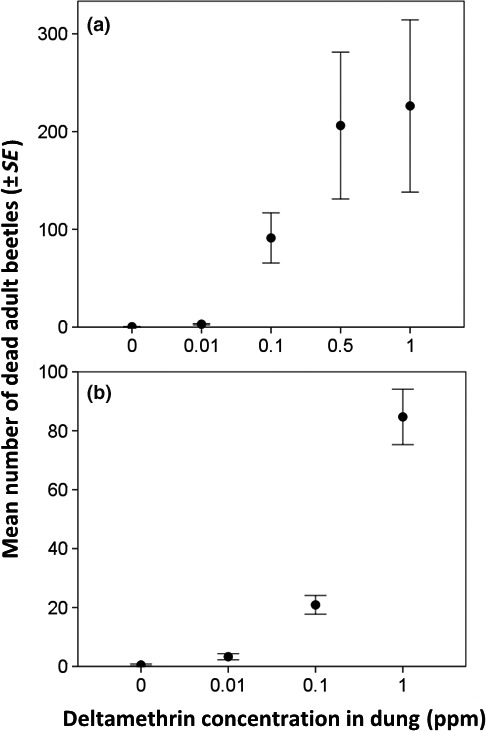
Mean (±*SE*) number of dead dung beetles found around pats containing different concentrations of insecticide in January to February (a) 2016 and (b) 2017

There were significant differences in the number of dead dung beetles found around the pats in both year 1 (*Z*_48_ = 4.82, *p *<* *.001; Figure 1a) and year 2 (*Z*_38_ = 6.20, *p *<* *.001; Figure 1b). Differences in mean beetle mortality between insecticide concentrations are presented in Table 1. In year 1 (2015/2016), there was a drought which may have contributed to differences in beetle abundances between years.

**Table 1:** Average (±*SE*) number of dead beetles found around pats containing different concentrations of deltamethrin in January–February 2016 and 2017. Within each year, different letters indicate a statistically significant difference in mean (*p* < .05)

Year	Deltamethrin concentration (ppm)				
	0	0.01	0.1	0.5	1
2016	0.4 ± 0.27^a^	3.0 ± 0.67^b^	91.2 ± 25.7^c^	206.2 ± 75.0^c^	226.2 ± 88.1^c^
2017	0.5 ± 0.31^w^	3.3 ± 1.02^x^	20.9 ± 3.18^y^	—	84.7 ± 9.40^z^

The majority of the dead beetles collected from around the pats were small paracoprids (<10 mm) in the tribe Onthophagini (97% in year 1 and 83% in year 2). Just 0.036% and 0.7% were large paracoprids in the tribe Coprini in years 1 and 2, respectively, and Aphodiinae comprised 2.4% of the total in year 1 and 16% in year 2.

### 3.2 Adult mortality—Bioassays

There was a significant interaction between deltamethrin concentration and exposure (*Z*_146_ = 3.178, *p *<* *.05); there was no significant relationship between beetle mortality and days exposure to insecticide‐free dung; and however, there was significant mortality over the 10 days of exposure to dung containing all insecticide concentrations: 0.01 ppm (*Z*_28_ = 2.72, *p *<* *.05), 0.1 ppm (*Z*_28_ = 5.58, *p *<* *.001), 0.5 ppm (*Z*_28_ = 4.15, *p *<* *.001), and 1 ppm (*Z*_28_ = 7.72, *p *<* *.001).

**Figure 2:**
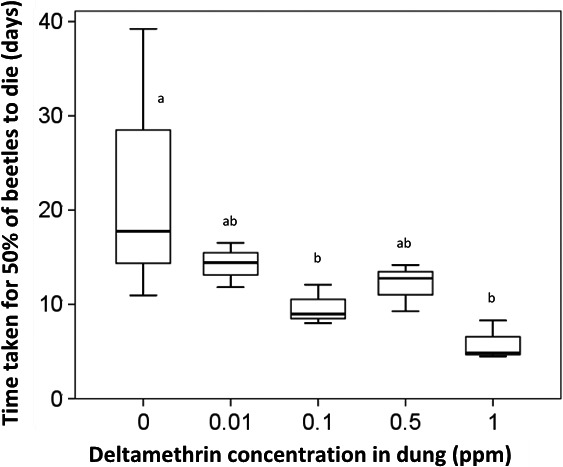
Time taken for 50% of adult *Metacatharsius troglodytes* (Boheman, 1857) to die (days) when exposed to dung containing different concentrations of deltamethrin. Boxes labeled with the same letters are not significantly different

The LT_50_ was significantly shorter for beetles exposed to 1 ppm (5.71 ± 0.66 days; *t*_10_ = −2.97, *p *<* *.05) and 0.1 ppm (9.44 ± 1.06 days; *t*_10_ = −2.30, *p *<* *.05) deltamethrin than to insecticide‐free dung (21.25 ± 17.35 days; Figure 2).

### 3.3 Reproduction

**Figure 3:**
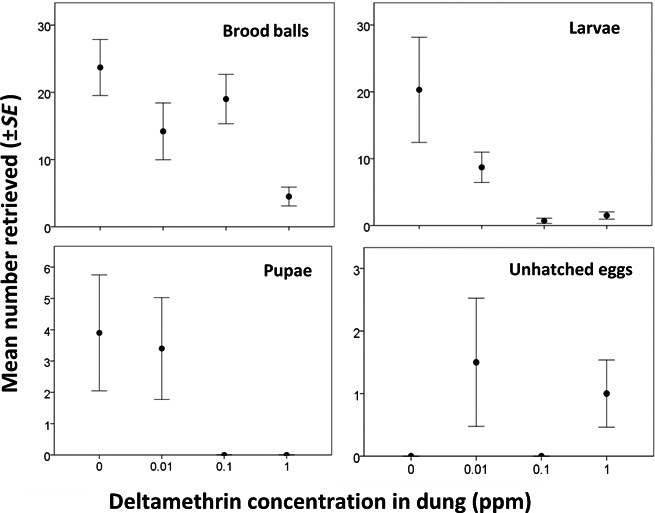
Mean number (±*SE*) of dung beetle (Scarabaeidae) brood balls, larvae, pupae, and unhatched eggs recovered from mesocosms containing dung of different deltamethrin concentrations

There was a significant difference in the number of brood balls (*Z*_38_ = −4.56, *p *<* *.001), larvae (*Z*_38_ = −3.33, *p *<* *.001), and pupae (*Z*_38_ = −2.31, *p *<* *.05) recovered from mesocosms with dung containing the different concentrations of deltamethrin. Post hoc comparisons using Tukey's HSD showed that there were significantly fewer brood balls produced from dung containing 1 ppm deltamethrin (mean 4.5 ± 1.4) than 0.1 ppm (*p *<* *.001; mean 19.0 ± 3.7), 0.01 ppm (*p *<* *.05; mean 14.2 ± 4.2), or insecticide‐free dung (*p *<* *.001; mean 23.7 ± 4.2; Figure 3). There were significantly fewer larvae found in mesocosms with 1 ppm (*p *<* *.001; mean 1.5 ± 0.5) and 0.1 ppm (*p *<* *.001; mean 0.7 ± 0.4) than insecticide‐free dung (mean 20.3 ± 7.8; Figure 3). No pupae were recovered from any of the mesocosms with dung containing 1 ppm or 0.1 ppm deltamethrin, whereas an average of 3.4 ± 1.6 and 3.9 ± 1.9 was recovered from 0.01 ppm and insecticide‐free dung, respectively (Figure 3). Unhatched eggs were found in brood balls that contained insecticide residues and not in insecticide‐free brood balls (Figure 3); however, the differences in the numbers between insecticide concentrations were not significant (*Z*_35_ = −0.59, *p *=* *.56).

### 3.4 Repellency

There were no significant differences in the number of beetles (subfamilies Aphodiinae and Scarabaeinae) attracted to insecticide‐free dung or dung containing any of the deltamethrin concentrations in either year 1 (*F*_7_ = 0.22, *p *=* *.83) or 2 (*F*_10_ = −0.91, *p *=* *.36).

### 3.5 Dung decomposition

**Figure 4:**
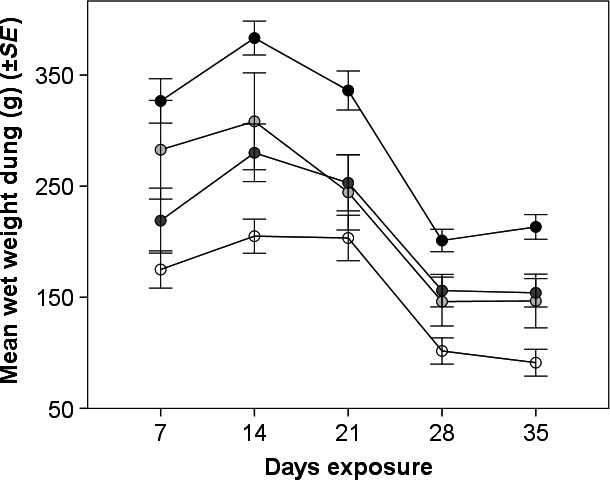
Mean wet wt (g; ±*SE*) standard 1 kg dung pats containing 0 ppm (white points), 0.01 ppm (light gray points), 0.1 ppm (dark gray points), and 1 ppm (black points) deltamethrin, measured weekly over 5‐week exposure in the field. Lines are joined for clarity

There was a significant reduction in dung weight over the 5 weeks (*F*_1,35_ = 112.6, *p *<* *.001; Figure 4). The fluctuation in wet weight of pats over time as a result of precipitation events was consistent between deltamethrin concentrations; that is, there was no significant interaction term between deltamethrin concentration and the number of days of field exposure. The weight of insecticide‐free pats was consistently significantly lower than that of pats made from dung containing any of the insecticide concentrations for the duration of the experiment (Table 2; Figure 4).

**Table 2:** Average wet wt (g) and *t* and *p* values for statistically significant differences in the mean wet wts between insecticide‐free control dung and dung pats containing different deltamethrin concentrations (0.01, 0.1, and 1 ppm; *N* = 10) on day 7, 14, 21, 28, and 35 after pat deposition. All pats weighed 1 kg on day 1

Day	Average (±*SE*) wet wt (g)				*t* Value			*p* Value		
	0 ppm	0.01 ppm	0.1 ppm	1 ppm	0.01 ppm	0.1 ppm	1 ppm	0.01 ppm	0.1 ppm	1 ppm
7	175 ± 17	274 ± 41	219 ± 29	327 ± 20	2.5	1.1	3.6	.016*	.29	.0011*
14	205 ± 15	298 ± 40	280 ± 26	383 ± 15	2.6	1.9	4.5	.013*	.06	<.001**
21	203 ± 21	244 ± 34	253 ± 25	336 ± 18	1.1	1.4	3.7	.26	.17	<.001**
28	102 ± 12	140 ± 21	156 ± 15	201 ± 10	2	2.5	4.5	.051*	.016*	<.001**
35	91 ± 12	147 ± 24	154 ± 13	213 ± 11	2.5	2.9	5.4	.020*	.0074*	<.001**

Significance codes: *p ≤ .05, **p < .001.

## 4 DISCUSSION

Impacts of deltamethrin on the dung beetle community colonizing cattle dung and the process of dung decomposition are reported here for an African pastoralist landscape. Concentrations were representative of those present in dung for up to 2 weeks following treatment of cattle with commercial topical formulations (Vale et al., 2004; Wardhaugh et al., 1998). Direct lethal effects were observed on adult Scarabaeidae; significantly more dead beetles were found around pats containing 0.01, 0.1 ppm, and 1 ppm deltamethrin than insecticide‐free pats. Mortality was predominantly seen in the small‐bodied Onthophagini paracoprid beetles, with larger bodied paracoprids such as Coprini comprising <1% of the total dead adults found. Other studies have also shown an accumulation of dead beetles in and around dung containing synthetic pyrethroid residues (Vale et al., 2004), and significantly fewer Scarabaeidae (adult, larval and pupal stages) were found colonizing pats that contained 0.01, 0.1, and 1 ppm deltamethrin compared to insecticide‐free control pats after 7 days of exposure in the field (Chihiya et al., 2006).

In the present work, the time taken for 50% of the paracoprid *M. troglodytes* to die (LT_50_) was significantly shorter for those exposed to dung containing 0.1 ppm and 1 ppm deltamethrin than insecticide‐free dung. Previous laboratory assays report lethal effects as a result of fecal contamination with a range of pyrethroid insecticides on many species of adult dung beetles (Scarabaeidae), mostly for dung collected during the first week after treatment. For example, dung collected from cattle up to 8 days after treatment with deltamethrin, cypermethrin, cyhalothrin, flumethrin, and alphamethrin has been shown to cause mortality in *Digitonthophagus gazella* (Fabricius 1787; Bianchin et al., 1992; Bianchin, Alves, & Koller, 1997; Bianchin et al., 1998). Treatment of cattle with spray‐on formulations of cis‐cypermethrin and chlorpyrifos resulted in significant mortality of *C. tripartitus* after 2 weeks of exposure to dung collected 1 day after treatment (Bang et al., 2007). The lethal concentration required to kill 50% (LC_50_) of adult Scarabaeinae was 0.01–1.82 ppm after 24‐hr exposure to dung spiked with pyrethroids (Vale et al., 2004). Inhibited dung consumption by *F*_2_ generation dung beetles (*Copris tripartitus* Waterhouse 1875) that were fed on dung from cattle treated with the synthetic pyrethroids cis‐cypermethrin and chlorpyrifos has also been noted (Bang et al., 2007). The present study confirms these findings in field conditions, with significant mortality of small‐bodied paracoprids at deltamethrin concentrations of 0.01 ppm and above.

Sublethal effects on reproduction were also evident under field conditions; there were significantly fewer brood balls produced from dung containing 1 ppm deltamethrin than control dung, and 20%–40% fewer brood balls were recovered from mesocosms with pats containing 0.01–0.1 ppm deltamethrin than from insecticide‐free pats. Previous laboratory assays have shown significantly fewer broods produced by *O. binodis* exposed to cattle dung collected 1, 3, 7, and 28 days after treatment with a deltamethrin pour‐on compared to dung from untreated cattle, but there was no effect on brood production by *Euoniticellus fulvus* (Goeze 1777; Wardhaugh et al., 1998). A significant reduction in *C. tripartitus* brood ball production was found after exposure to dung collected one day after treatment with cis‐cypermethrin and chlorpyrifos, but no effect was seen for dung collected 3, 5, or 7 days after treatment (Bang et al., 2007). However, prolonged exposure to dung collected 3 and 5 days after treatment resulted in significantly reduced numbers of brood balls being produced the following year. These experiments imply varying sensitivities of dung beetle species to pyrethroid formulations. The present study considered the whole dung beetle community at the field scale and indicates that while fewer brood balls are produced at 0.01–0.1 ppm deltamethrin, dung beetles do still bury dung containing insecticide residues. Over a large agricultural area and for sustained periods however, even a small reduction in brood production may be ecologically important.

When developing underground in contaminated brood balls, significantly fewer dung beetle larvae and pupae were recovered from 0.1 and 1 ppm deltamethrin than insecticide‐free control dung. A field study in the UK reported a complete absence of Scarabaeidae larvae and pupae in pats from cows treated 1, 3, and 5 days previously with a pour‐on deltamethrin product (Mann et al., 2015). In the present work, unhatched eggs were found inside brood balls made from dung containing 0.01 and 1 ppm deltamethrin, and virtually, no larvae were present in brood balls made from dung containing 0.1 ppm, whereas many healthy larvae were found in insecticide‐free brood balls. Further work is required to provide conclusive data regarding the possible ovicidal effects of deltamethrin residues in brood balls. Altogether, these sublethal effects on reproduction suggest that even if dung beetle populations appear unaffected within a season, and dung is still being removed from the pasture surface, over several generations and under repeated insecticide pressure, the insecticidal activity on the juvenile stages below the soil surface is likely to be detrimental to dung beetle species and population abundance in an area.

No repellent effects were observed for deltamethrin concentrations of 0.01, 0.1, or 1 ppm in dung. To date, there are no previous studies published that specifically examine repellent effects of pyrethroids on dung beetles, although repellency has been suggested by some authors (Bianchin et al., 1998; Mann et al., 2015; Vale et al., 2004). The data from the repellency study presented here suggest that there is no immediate repellent effect of deltamethrin residues in the dung. However, field observations implied that dung beetles may colonize the dung as normal, but toxic effects then cause them to leave the pats and die; many dead and moribund beetles were found on the soil surface in the area around contaminated pats. Observations by Vale et al. (2004) support this notion; it was found that the edge of contaminated pats often becomes “shredded” due to beetles burrowing in and back out. This could result in the dung of treated livestock forming an ecological trap, whereby the habitat is degraded by the presence of insecticide, but the cues normally correlating with a high‐quality habitat still exist (Dwernychuk & Boag, 1972; Schlaepfer, Runge, & Sherman, 2002).

Dung decomposition was significantly reduced by the presence of deltamethrin at all time points measured (7, 14, 21, 28, and 35 days postpat deposition). Wet weights were used as they allowed repeated nondestructive sampling (Beynon, Mann, Slade, & Lewis, 2012), and as earthworms were absent from this semiarid African ecosystem, there were no inaccuracies in measurement resulting from mineral soil in their casts in the dung (Holter, 1977). Although within‐group dung weights fluctuated over time depending on recent precipitation events, between‐group differences in weight could clearly be observed. Even at the lowest deltamethrin concentration (0.01 ppm), the weight of dung remaining on the soil surface was significantly higher than insecticide‐free control dung for the duration of the experiment. A previous study in Zimbabwe similarly reported reductions in decomposition at 10 and 100 days postpat deposition; however, effects were only significant for dung concentrations of 0.1 ppm deltamethrin and above (Vale et al., 2004). Others have found a significantly reduced loss of organic matter in dung from cattle treated with α‐cypermethrin compared to untreated control dung over 12 and 16 weeks in the field in Denmark (Sommer & Bibby, 2002), suggesting that the process of decomposition is affected over longer periods in a temperate system. The results of the present study suggest that the lethal and sublethal effects of pyrethroid residues on dung fauna in a southern African ecosystem are sufficient to significantly inhibit their provision of the ecosystem service of dung decomposition.

The data presented here indicate that the widespread use of pyrethroid insecticides in African agricultural ecosystems is likely to be a significant cause for concern. The combined effects on survival and reproduction are such that dung beetle populations are likely to decline if subjected to sustained and widespread pyrethroid use. A reduction in the ecosystem services provided by dung beetles, including dung decomposition, bioturbation, nutrient cycling, pasture fertility, and livestock parasite suppression (Bang et al., 2005; Barth, Heinze‐Mutz, Roncalli, Schlüter, & Gross, 1993; Nichols et al., 2008; Sands & Wall, 2016), could prevent ecosystem function in agricultural landscapes resulting in reduced productivity and economic loss for farmers (Beynon et al., 2015). In addition, there could be broader ecological impacts on the insect, mammal, bird, and reptile populations which rely on dung beetles as prey items (Young, 2015).

Recommendations for increasing the environmental and economic sustainability of livestock pest and parasite management are likely to include choosing compounds and formulations that are known to be less harmful to dung beetles (Bianchin et al., 1992; Kryger, Deschodt, Davis, & Scholtz, 2006, 2007), or treating only certain parts of the body surface where parasites prefer (Vale, Hargrove, Chamisa, Grant, & Torr, 2015), for example the legs and belly in the case of tsetse flies (Torr, Maudlin, & Vale, 2007). Staggered treatment of animals to ensure the presence of insecticide‐free refugia pats on pastures at all times targeted treatment of affected individuals, and integrated approaches may also help to minimize damage (Wall & Beynon, 2012).

## CONFLICT OF INTEREST

None declared.

## AUTHOR CONTRIBUTIONS

BS and RW conceived the ideas and designed the methodology; BS, NM, and CN collected the data; BS analyzed the data; BS and RW led the writing of the manuscript. All authors contributed critically to the drafts and gave their final approval for publication.

## DATA ACCESSIBILITY

Experimental data: Dryad Digital Repository.
